# An Intelligent Failure Detection on a Wireless Sensor Network for Indoor Climate Conditions

**DOI:** 10.3390/s19040854

**Published:** 2019-02-19

**Authors:** Sebastián Gutiérrez, Hiram Ponce

**Affiliations:** 1 Facultad de Ingeniería, Universidad Panamericana, Josemaría Escrivá de Balaguer 101, Aguascalientes, Aguascalientes 20290, Mexico; jsgutierrez@up.edu.mx; 2 Facultad de Ingeniería, Universidad Panamericana, Augusto Rodin 498, Ciudad de México 03920, Mexico

**Keywords:** artificial organic networks, artificial hydrocarbon networks, distributed services architecture, failure detection, internet-of-things, machine learning, weather web services, sensor networks

## Abstract

Wireless sensor networks (WSN) involve large number of sensor nodes distributed at diverse locations. The collected data are prone to be inaccurate and faulty due to internal or external influences, such as, environmental interference or sensor aging. Intelligent failure detection is necessary for the effective functioning of the sensor network. In this paper, we propose a supervised learning method that is named artificial hydrocarbon networks (AHN), to predict temperature in a remote location and detect failures in sensors. It allows predicting the temperature and detecting failure in sensor node of remote locations using information from a web service comparing it with field temperature sensors. For experimentation, we implemented a small WSN to test our sensor in order to measure failure detection, identification and accommodation proposal. In our experiments, 94.18% of the testing data were recovered and accommodated allowing of validation our proposed approach that is based on AHN, which detects, identify and accommodate sensor failures accurately.

## 1. Introduction

Nowadays, weather conditions affect the daily life of many people in the world [1]. The need of environmental information for the development of activities around the cities, farms and communities is increasing as technology of meteorological predictions becomes available. Likewise, the grown of development of meteorological stations for reports is increasing [2,3,4,5,6,7]. The weather monitoring stations help to predict and understand the weather conditions in order to monitor and track weather changes. These stations uses multiple meteorological sensors to monitoring weather conditions by sensing weather variables such as temperature, humidity, atmospheric pressure, wind speed, wind direction and rainfall, with the objective to give accurate information to users.

In fact, some institutions and companies, and in some cases, buildings and homes, have their own weather stations to collect weather data. With a right distribution of meteorological sensors, a prediction based on acquired data with information obtained in real time can be created. This information is used to their own ends, and in some cases, shared between third parties through a new rising technology like the Internet of Things (IoT) to keep in hand the needed information to anticipate and be alert to the weather and climate conditions.

In this context, IoT is a new part of a novel generation of information technology that allows the connection between devices to share multiple data to control, automate and centralize systems of a variety of application domains. This technology has several application domains such as environmental monitoring, smart cities, smart business and product management, smart homes, smart building management, healthcare and security and surveillance. To achieve this, many protocols for communication and management must allow the connection with sensors, cloud-based systems, control devices and monitoring [8,9,10]. Moreover, it is important to mention that the failures from sensor nodes to receive messages must be taken into account, i.e., sensors can have a poor connection to the base station, meaning that the data transmission from that sensor has a high risk to fail. In addition, due to the limited energy capacity, sensor nodes with run out battery may cause disconnected links which will result in failed data transmission in IoT [11,12,13].

The implementation of weather monitoring station based on IoT system allows to obtain information from weather web services and physical multi-sensors connected into IoT network, attaining better accuracy of data used to predict and show weather statistics [14,15,16,17]. Moreover, the problem of failure detection in sensor nodes is essential since this issue can destabilize the normal operation of measurement. In addition, artificial intelligence (AI), i.e., machine learning and data mining, enhance predictive climate conditions actions over IoT systems [18]. And, some approaches have been developed on sensor failure using AI.

In particular, sensor failure detection, identification and accommodation (SFDIA) approaches are those schemes used to preserve robustness and high reliability of sensor-based systems [19]. For instance, sensor accommodation (SA) refers to the ability of the system to replace the malfunctioning sensor with another sensor or estimator. A typical scheme for SA is redundancy in hardware using multiple sensors for one measurement, in which, if one of them has different behavior than the others, one of the other sensors replaces it [19]. Sensor failure detection and identification (SFDI) refers to determine an anomaly in the system and to identify where it takes place. Literature reports SFDI schemes is mainly applied with the comparison between the real sensor value and an estimation of it [19,20]. For estimation, linear and nonlinear models have been applied [19]. Recently, AI has been applied to solve SFDIA systems. Another approach, mainly for wireless sensor networks, is the check-point recover algorithm which measures the energy level of a sensor and sends that information through all the network. If one sensor is not able to send this energy level, then it is assumed as a fault [21]. In literature [19,20,21,22], some proposal for failure detection in sensors through neural networks learning algorithms and support vector machines have been reported.

In this context, this paper proposes to apply artificial intelligence by means of a supervised learning method namely artificial hydrocarbon networks (AHN), to predict temperature in a remote location and detect failures in sensors. For this work, a small wireless sensor network over IoT protocols is developed. This network consists on three temperature sensors, remotely located indoors, and a temperature web service. At each node of the network, an AHN-model for predicting temperature is trained, but also the architecture of the AHN considers a failure detection method. In this sense the system is able to identify a failure in sensors over the wireless network and to promote SFDIA. For validation purposes, a simulated and a real data sets of large number of sensor nodes were previously tested.

The contribution of this work considers: (i) the development of a wireless sensor network for indoor temperature estimation using AHN, and (ii) the proposed sensor failure detection module using AHN. To the best of our knowledge, this is the first time AHN is implemented for failure detection systems.

The rest of the paper is organized as follows. Section 2 summarizes the prototype development of the IoT system. Section 3 describes the proposed intelligent sensor failure detection module using artificial hydrocarbon networks. Section 4 shows the applicability of the proposed strategy in large wireless sensor networks. Section 5 presents experimental results and discussion about the proposal, and Section 6 concludes this work.

## 2. Prototype Development

**Figure 1:**
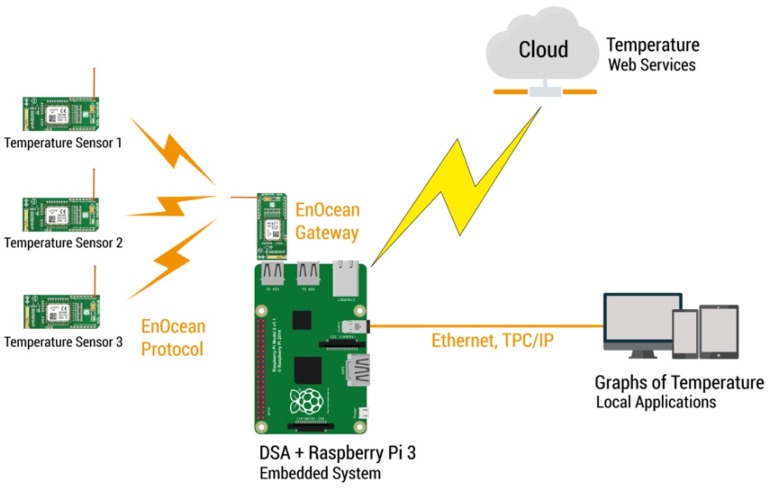
Architecture of the IoT system.

The prototype implementation of the proposed system uses a weather web service and physical temperature sensors under DSA (Distributed Services Architecture) open source platform for IoT [23]. The main goal of the DSA is to unify multiple devices, services and application in real time, structured and adaptable data model. To achieve this, we employed an embedded system Raspberry Pi 3 as a DSA server to control the system using a distributed server link (DSLink) to enable data exchange with other connected sensor nodes, as a weather web service in the cloud that uses Yahoo Weather API [24], and a communication protocol for receiving physical sensors data by means of EnOcean®protocol [25,26,27]. Figure 1 shows the IoT architecture of the prototype development.

In addition to the embedded system, the DGLux5®program, integrated in the DSA server, allows to work with real time data to ensure the design and deployment process can be shown satisfactory on the different temperatures from physical sensors and the weather web service. The visual programming language to designing the front-end was performed connecting blocks that represent different operations. In the dataflow, to display the various temperatures and save the values in a database, the blocks were dragged and placed to be connected.

**Figure 2:**
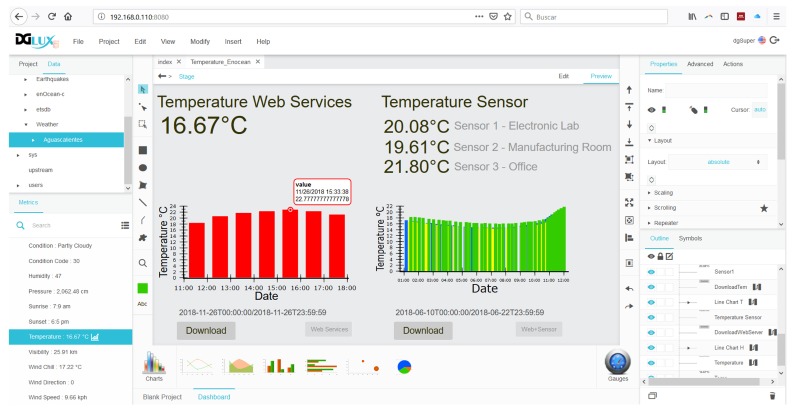
Snapshot of the front-end developer of DGLux5 ®program.

As shown in Figure 1, the DSA obtains the temperatures from the weather web service connected to the cloud via internet and collects the information from the physical sensors using the EnOcean®protocol. The temperature data obtained are shown in a graphical interface of DGLux5®program, where each of the temperatures appears, and where it is possible to download a CSV file of each one of them, as shown in Figure 2.

**Figure 3:**
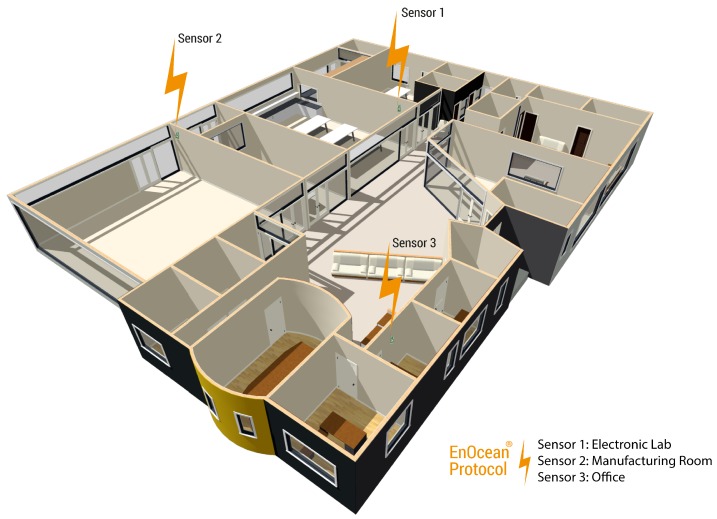
Layout of the building showing the location of the three temperature sensors.

**Figure 4:**
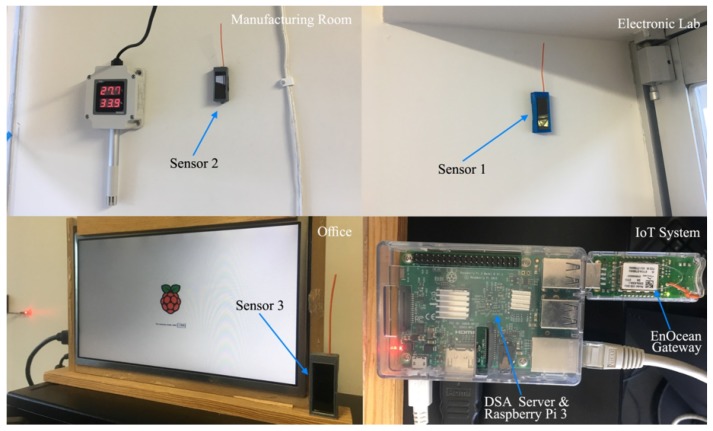
Location of the three temperature sensors inside enclosures.

For this prototype implementation, the temperature sensors were located physically in three different locations inside a building of the Faculty of Engineering in Universidad Panamericana (Aguascalientes, Mexico). Sensor 1 was located on an electronic laboratory; sensor 2 was placed in a manufacturing room; and finally, sensor 3 was positioned on an office. Figure 3 shows the locations of the three sensors inside the building, while Figure 4 shows the inside enclosures where the sensors were placed.

## 3. Development of the Temperature Estimation and Sensor Failure Detection System

**Figure 5:**
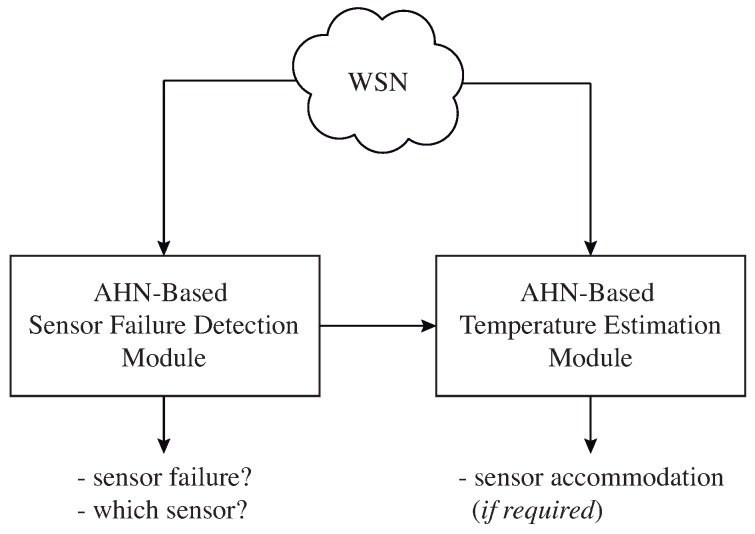
Proposed intelligent system for temperature estimation and sensor failure detection.

In order to perform an intelligent system that can predict temperature changes and failure detection in sensors through an IoT system, the AHN supervised learning method is proposed. In particular, this work is focused solely on the prediction of the temperature and failure detection sensors using the IoT system presented in Section 2. The proposed intelligent system is shown in Figure 5. The ensuing is a general description of wireless sensor network architecture, the method of artificial hydrocarbon networks and the description of the intelligent module.

### 3.1. Wireless Sensor Networks

**Figure 6:**
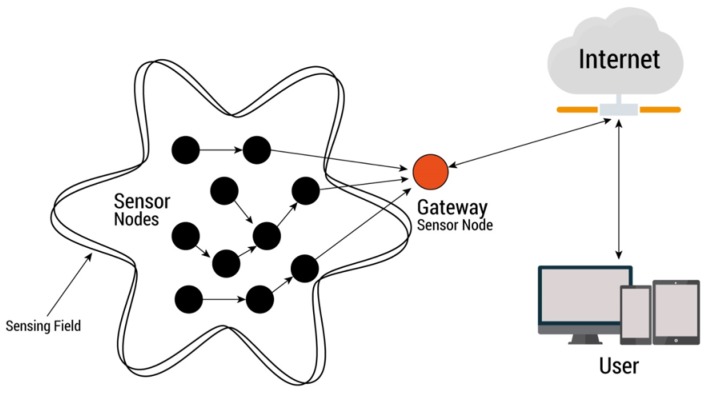
Wireless sensor network architecture of the IoT system.

A wireless sensor network (WSN) is composed of a large number of sensor nodes that cover an even larger set of application areas [28], such as, military and surveillance [29,30,31], environment [3,32,33], health [34,35,36], home and building monitoring [37,38,39]. On the network, sensor nodes send information at regular time intervals from inside enclosures to be monitored, and ensure that the nodes in the network operate faultlessly. Therefore, fault detection in sensors nodes play an important role in network monitoring since an error can have a negative effect on the system’s efficiency and reliability. Figure 6 shows our WSN architecture that is applied in temperature monitoring which contains sensor nodes, gateway and user. Sensor nodes will communicate with each other and transmit the processed data to gateway over a wireless communication. The gateway collects data from all the sensor nodes, transmits the analyzed data to user via Internet and receives data from temperature web services.

### 3.2. Artificial Hydrocarbon Networks

In machine learning, AHN is a supervised learning method. It aims to model data using the inspiration of carbon networks, simulating the chemical rules involved within organic molecules to represent the structure and behavior of data [40,41]. Thus, the method loosely simulates the chemical interactions of hydrocarbon molecules.

**Figure 7:**
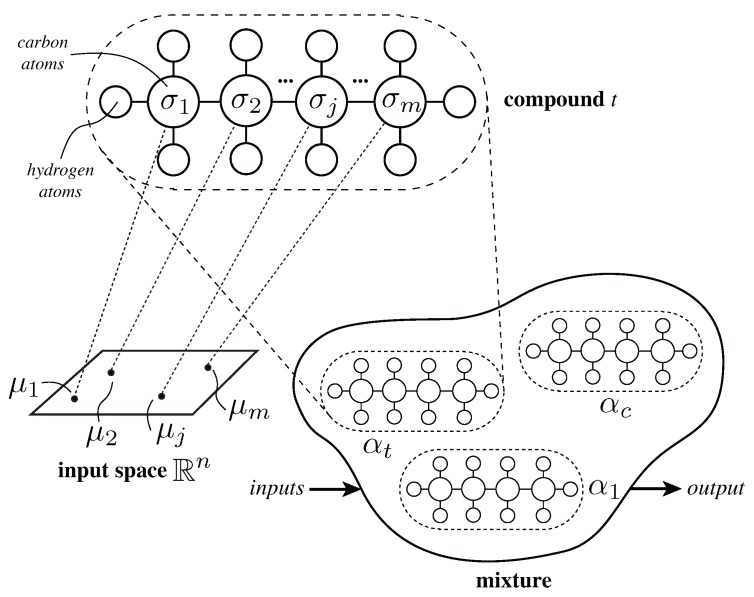
Schematic of an artificial hydrocarbon network.

The basic unit of information in AHN is namely CH-molecule or simply molecule. From inspiration on nature, a molecule is formed with one carbon atom that is linked to up to four hydrogen atoms. Generally speaking, one molecule models a chunk of data in its parameters (hydrogen and carbon atoms) and configuration. The molecule has a structural representation, a graphical model as shown in Figure 7, and a chemical behavior. Mathematically, the behavior φ of a molecule with *k* hydrogen atoms is expressed as in (1); where, $\sigma \in R^{n}$ is called the carbon value, $H_{i}\in R^{n}$ is the *i*-th hydrogen atom attached to the carbon atom, and $x=(x_{1},\cdots ,x_{n})$ is the input vector with *n* features.

(1) $$\varphi \left(x,k\right)=\sum_{r=1}^{n}\sigma_{r}\sum_{i=1}^{k\leq 4}H_{ir}x^{k}$$

Two or more unsaturated molecules, i.e., those with k<4 hydrogen atoms, can be joined together. In AHN, this structure is called a compound. Different compounds have been defined in literature [40,42,43]. The simplest one is the saturated and linear chain of *m* molecules. It is composed structurally of two molecules with three hydrogen atoms and (m−2) molecules with two hydrogen atoms. The behavior ψ of a saturated-and-linear compound is defined as (2); where, $\varphi_{j}$ is the behavior of the *j*th associated molecule that represents a subset $\Sigma_{j}$ of the input *x* such that $\Sigma_{j}=\left\{x|\arg\min_{j}\left(x-\mu_{j}\right)=j\right\}$, and $\mu_{j}\in R^{n}$ is the center of the *j*th molecule [44,45]. In fact, $\Sigma_{j_{1}}\cap \Sigma_{j_{2}}=\emptyset$ if $j_{1}\neq j_{2}$. In this regard, literature reports extensive usage of AHN with a single saturated-and-linear compound [40,41,43,44,45,46].

(2) $$\psi \left(x\right)=\left\{\begin{array}{cc} \varphi_{1}\left(x,3\right) & x\in \Sigma_{1}\\ \varphi_{2}\left(x,2\right) & x\in \Sigma_{2}\\ \cdots & \cdots \\ \varphi_{m-1}\left(x,2\right) & x\in \Sigma_{m-1}\\ \varphi_{m}\left(x,3\right) & x\in \Sigma_{m} \end{array}\right.$$

Compounds can interact among them in definite ratios $\alpha_{t}$, so-called stoichiometric coefficients or weights, forming a mixture S(x) as shown in (3); where, *c* represents the number of compounds in the mixture and $\alpha_{t}$ is the weighted factor of the *t*-th compound [40].

(3) $$S\left(x\right)=\sum_{t=1}^{c}\alpha_{t}\psi_{t}\left(x\right)$$

Formally, an AHN is a mixture of compounds (see Figure 7) each one computed using a chemical-based heuristic rule, expressed in its own training algorithm, as depicted in Algorithm 1 [40,42,46]. At first, it initializes the structure of a saturated and linear compound with *m* molecules, and the centers of molecules $\left\{\mu_{j}\right\}$ are randomly set. While a stop criterion is not reached, the compound is computed and updated as follows. First, for each molecule, the training dataset Σ is partitioned in subsets $\Sigma_{j}$ such that every input *x* is near to $\mu_{j}$. Then, the hydrogen and carbon values of each molecule is computed independently using the least squares estimates (LSE) method, and the error $E_{j}$ between the output response of the *j*-th molecule and the actual targets *y* of the *j*-th subset is calculated. If any of the subsets is empty, then these subsets require a new position, i.e., update their centers. This relocation is made by simply changing the center of the empty subset randomly close to one molecule with large error. Later on, the center of molecules are updated via a gradient descent approach with learning rate 0<η<1, previously chosen, as shown in (4) with $E_{0}=0$. Lastly, the compound ψ is updated with the behaviors of molecules calculated so far.

(4) $$\mu_{j}\leftarrow \mu_{j}-\eta \left(E_{j-1}-E_{j}\right)$$

A detailed description of the AHN-algorithm can be found in [40]. Information about properties and comparisons with other supervised learning methods can be seen in [40,44,45].

### 3.3. Temperature Estimation and Sensor Failure Detection Module

For our proposal, we model the dynamics of the temperature sensors in the WSN to provide an estimate using AHN. In addition, this estimation is then compared with the actual sensor values, and treated statistically for failure detection. In the following, the entire module is explained.

**Algorithm 1** Simple AHN training algorithm

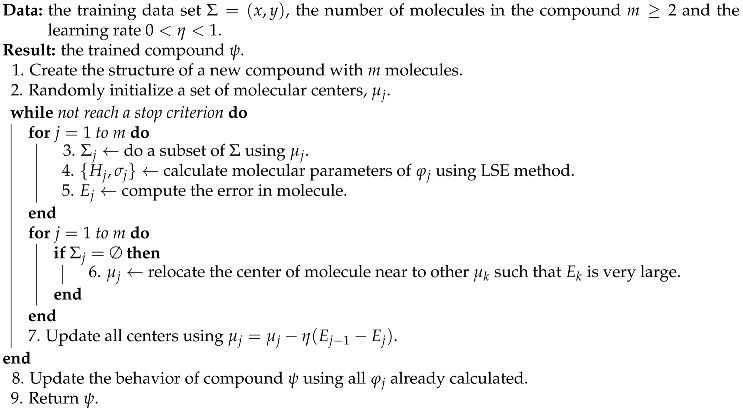



#### 3.3.1. Temperature Estimation Module

Consider a WSN with *N* sensors as nodes, $s_{i}$ for all i=1,⋯,N. Also, let say that sensor $s_{i}$ fails. Then, we propose a sensor accommodation using the estimations of an AHN-based model ${\hat{fs}}_{\omega }^{\left(i\right)}$ of sensor $s_{i}$, as expressed in (5); where, ${\hat{s}}_{i}\left(t\right)\in R$ is the estimation of sensor $s_{i}$ in time *t* and $s_{wsn}\left(t\right)\in R^{\left(N-1\right)}$ is a vector of values of the (N−1) sensors $s_{wsn}\left(t\right)=\left(s_{1}\left(t\right),\cdots ,s_{k}\left(t\right),\cdots ,s_{\left(N-1\right)}\left(t\right)\right)$ and i≠k in time *t*.

(5) $${\hat{s}}_{i}\left(t\right)={\hat{fs}}_{\omega }^{\left(i\right)}\left(s_{wsn}\left(t\right),s_{wsn}\left(t-1\right)\right)$$

This sensor model ${\hat{fs}}_{\omega }^{\left(i\right)}$ is proposed to be based on AHN with a vector of parameters ω that represents the hydrogen, carbon and molecular centers over the hydrocarbon compound, such that $\omega =\{H_{ir},\sigma_{r},\mu_{j}\}$. For training the AHN-model, we use tuples of the form $\left(s_{wsn}\left(t\right),s_{wsn}\left(t-1\right)\right)\in R^{2(N-1)}$ as training inputs and $s_{i}\left(t\right)\in R$ as training outputs, collected from the WSN for a given period of time. In this regard, the sensor model ${\hat{fs}}_{\omega }^{\left(i\right)}$ is trained by minimizing the error function described in (6) using the gradient descent method.

(6) $$E\left(\omega \right)=\frac{1}{2}\sum_{s_{wsn}\left(t\right),s_{wsn}\left(t-1\right)\in R^{2*(N-1)}}{∥s_{i}\left(t\right)-{\hat{fs}}_{\omega }^{\left(i\right)}\left(s_{wsn}\left(t\right),s_{wsn}\left(t-1\right)\right)∥}^{2}$$

#### 3.3.2. Failure Detection Module

The aim of this module is to detect sensor failures through implicit estimations of the dynamic system. In general, this process considers two steps: (i) compute the error between the dynamic of the system and the estimates, and (ii) compute a metric for failure detection. This is described below.

Consider a WSN with *N* sensors as nodes, $s_{i}$ for all i=1,⋯,N. Moreover, consider the dynamics function model ${\hat{f}}_{\omega }$ of a sensor as (7), where $s_{i}\left(t\right)\in R$ is the value of sensor $s_{i}$ in time *t*, $s_{wsn}\left(t\right)\in R^{\left(N-1\right)}$ is a vector of values of the (N−1) sensors $s_{wsn}\left(t\right)=\left(s_{1}\left(t\right),\cdots ,s_{k}\left(t\right),\cdots ,s_{\left(N-1\right)}\left(t\right)\right)$ and i≠k in time *t*, and $\Delta {\hat{s}}_{wsn}\left(t\right)$ represents the changes in sensors values $s_{wsn}$ in time *t*.

(7) $$\Delta {\hat{s}}_{wsn}\left(t\right)={\hat{f}}_{\omega }\left(s_{wsn}\left(t\right),s_{i}\left(t\right)\right)$$

The dynamic function ${\hat{f}}_{\omega }$ is proposed to be parameterized with AHN, where the vector of parameters ω represents the hydrogen, carbon and molecular centers over the hydrocarbon compound, i.e., $\omega =\{H_{ir},\sigma_{r},\mu_{j}\}$. After training the AHN-model ${\hat{f}}_{\omega }$ and using (7), it is easy to compute the predicted next values of sensors $s_{wsn}$ for time t+1 as expressed in (8).

(8) $${\hat{s}}_{wsn}\left(t+1\right)=s_{wsn}\left(t\right)+{\hat{f}}_{\omega }\left(s_{wsn}\left(t\right),s_{i}\left(t\right)\right)$$

For training the AHN-model, we use tuples of the form $\left(s_{wsn}\left(t-1\right),s_{i}\left(t-1\right)\right)\in R^{N}$ as training inputs and differences $\Delta s_{wsn}\left(t\right)=s_{wsn}\left(t\right)-s_{wsn}\left(t-1\right)\in R^{N-1}$ as training outputs, collected from the WSN for a given period of time, and all these data were randomly shuffled. Thus, we train the dynamics function model ${\hat{f}}_{\omega }$ by minimizing the error (9) using the gradient descent method.

(9) $$E\left(\omega \right)=\frac{1}{2}\sum_{s_{wsn}\left(t-1\right),s_{i}\left(t-1\right)\in R^{N}}{∥\Delta s_{wsn}\left(t\right)-{\hat{f}}_{\omega }\left(s_{wsn}\left(t-1\right),s_{i}\left(t-1\right)\right)∥}^{2}$$

It is remarkable to say that using the definition from (7), it is possible to observe that changes of sensors values $\Delta {\hat{s}}_{wsn}$ in *t* are impacted by the actual values of the sensor $s_{i}\left(t\right)$. Thus, if $s_{i}\left(t\right)$ fails, then it will impact on the changes of sensors values and also in the prediction of the next values ${\hat{s}}_{wsn}\left(t+1\right)$.

From the above, it is possible to detect a failure in the system comparing the actual values of sensors $s_{wsn}\left(t\right)$ and the estimation ${\hat{s}}_{wsn}\left(t\right)$ as shown in (10), where $E_{i}$ is the error between the actual and estimate values of the sensors using the function model excited from $s_{i}$.

(10) $$E_{i}\left(t\right)=∥s_{wsn}\left(t\right)-{\hat{s}}_{wsn}\left(t\right)∥$$

Since an actual failure of $s_{i}$ affects on all the function models built for each sensor in the WSN, then $E_{j}$ for all j=1,⋯,N are affected differently. To take into account all these variations, an overall error (11) is computed.

(11) $$E\left(t\right)=\sum_{j=1}^{N}E_{j}\left(t\right)$$

It would be easy to use a threshold scheme for sensor failure detection [19]. However, tuning the threshold is highly dependent on the behavior of the WSN. Hence, we propose to normalize the overall error online, obtaining its mean $\mu_{E}$ and standard deviation $\sigma_{E}$ from zero to *t*, such a confidence value α for failure detection can be selected. To this end, the failure detection function fail(t) can be expressed as in (12).

(12) $$fail\left(t\right)=\left\{\begin{array}{cc} 1 & {\left(\frac{E\left(t\right)-\mu_{E}}{\sigma_{E}}\right)}^{2}>\alpha \\ 0 & otherwise \end{array}\right.$$

#### 3.3.3. Sensor Identification

The aim of this phase is to determine which sensor fails in a given time *t*. From the above description, if a sensor failure is produced, then it is possible to detect it comparing the dynamic system with the estimation of the dynamics. Since the estimation dynamics is computed for each sensor node, then the one with the highest error would be identified as the failure one, as described below.

To identify a failed sensor $s_{i}$ over a WSN of *N* sensor nodes, it is possible to simply compare the actual values $s_{k}\left(t\right)$ and the estimation values ${\hat{s}}_{k}\left(t\right)$ of all sensors (using the sensor model ${\hat{fs}}_{\omega }^{\left(k\right)}$), and determine which sensor *k* obtains the maximum difference, as calculated in (13).

(13) $$i=\max_{k}∥s_{k}\left(t\right)-{\hat{s}}_{k}\left(t\right)∥$$

#### 3.3.4. SFDIA Using AHN

To this end, a complete SFDIA is proposed over a WSN of *N* sensor nodes by employing AHN at different levels of the approach. Algorithm 2 summarizes the whole SFDIA strategy.

It consists in five phases: (i) a training step that consists on collecting healthy data for building all AHN-models required for the strategy (sensor models and dynamic function models), (ii) a failure detection step that determines if there exists or not a sensor failure, (iii) a sensor identification step where the system recognizes which sensor failed, (iv) a sensor accommodation step that replaces the sensor failed by the sensor estimations done with its related sensor model, and (v) an update statistics step that recalculates the statistical values of overall error that allows to define a failure (c.f. Figure 5).

**Algorithm 2** Sensor failure, identification and accommodation strategy using AHN

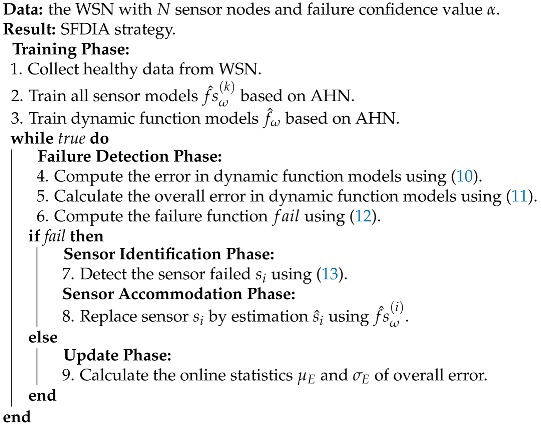



## 4. Applicability of the Proposed SFDIA with AHN

We conducted a preliminary experimentation using the SFDIA strategy including AHN in order to test its performance over large WSN. Two experiments using both simulated and real WSN were done, as described in the following section.

### 4.1. SFDIA with AHN on Simulated WSN

**Figure 8:**
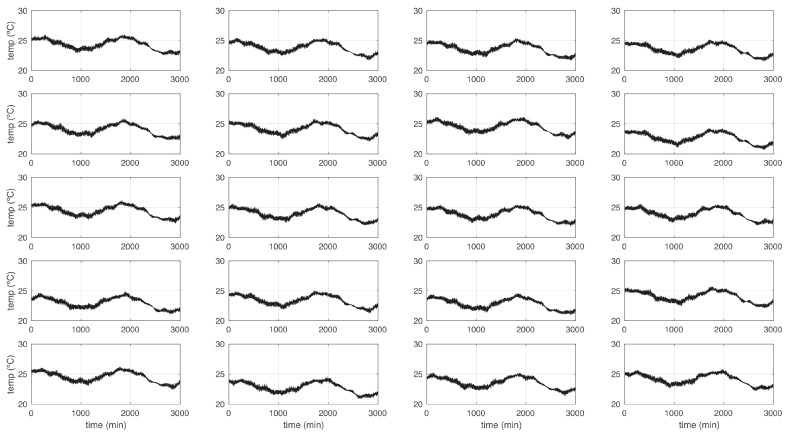
Thumbnails of sensor nodes in a simulated WSN with N=20.

We simulated three WSN with different number of nodes, ranging from N=20,50 and 100. In this case, each sensor node simulates the measurement of indoor temperature in different locations. Data were simulated using a pseudo-stochastic periodic function and random values in the frequency and translation parameters. Figure 8 shows thumbnails of sensor nodes in the experiment with N=20, only in the testing data.

For training sensor models and dynamic function models, we generated 10,000 samples, 70% of them for training and 30% for testing. The training set was considered as healthy information. We built all AHN models using the following parameters: molecules m=5, learning rate η=0.1 and a maximum of 1000 iterations as stop criterion. For suitability, data were normalized uniformly before using at training step.

**Figure 9:**
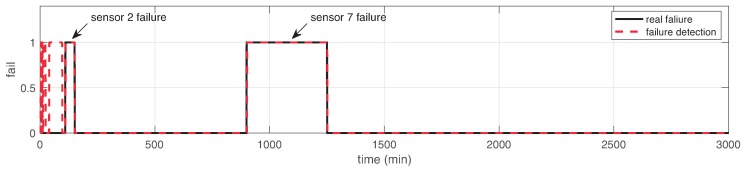
Failure detection response in the system, for N=20.

We then polluted sensor data artificially during two windows of time. These windows were chosen to be fixed in positions for comparative purposes. The windows for failure sensors were in ranges (110,150) min and (900,1250) min. After applying our proposed SFDIA strategy, of Algorithm 2, over the different scenarios (N=20,50,100) in WSN, we obtained the results summarized in Table 1. As shown, these results report the accuracy of the failure detection phase, using (12), in comparison with the ground-truth. Figure 9 depicts an example when N=20 of the comparison between the estimated failure detection and the real failure.

It is shown in Figure 9 that the first part of the estimation in failure detection computes several false-positive. However, this is not an issue assuming that in these regions, the actual values of the identified failure sensor will be replaced by estimated values using the corresponding sensor model. Moreover, from Table 1 it can be observed that increasing the number of nodes does not affect significantly the accuracy of failure detection, e.g., mean accuracy of 89.42%. Thus, our proposed SFDIA with AHN can deal with different number of sensor nodes in WSN.

### 4.2. SFDIA With AHN on Real WSN

**Figure 10:**
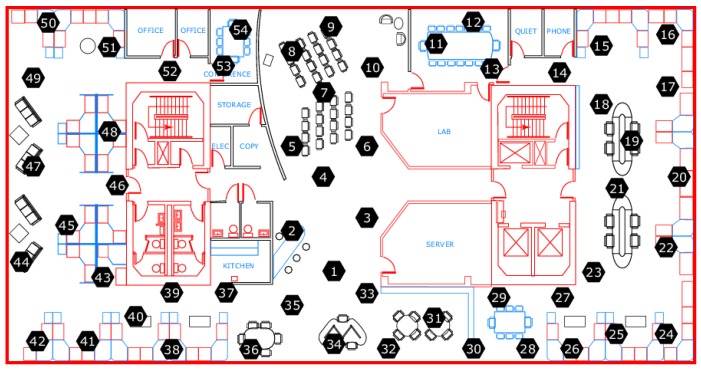
Location of the sensor nodes in the Intel Lab. Layout taken from [47].

We tested our proposed SFDIA with AHN on a real WSN. We employed the Intel Lab data set [47] that comprises 54 sensors retrieving weather data such as: temperature, relative humidity and light, as well as the voltage of the battery. Data were gathered in the Intel Lab, as shown in Figure 10, between 28 February and 5 April 2004.

**Figure 11:**
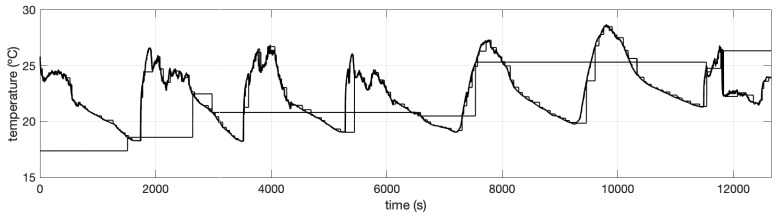
Training data set obtained from the Intel Lab WSN with N=54 sensor nodes.

For training sensor models and dynamic function models, we extracted 12651 samples for training and 22,251 for testing. These data only contain temperature values as continuous time series. The training set was considered as healthy information, as depicted in Figure 11. In this case, testing data contain altered values (e.g., far from the mean value of time series) from the original data set. We built all AHN models using the following parameters: molecules m=5, learning rate η=0.1 and a maximum of 1000 iterations as stop criterion. For suitability, data were normalized uniformly before using at training step.

**Figure 12:**
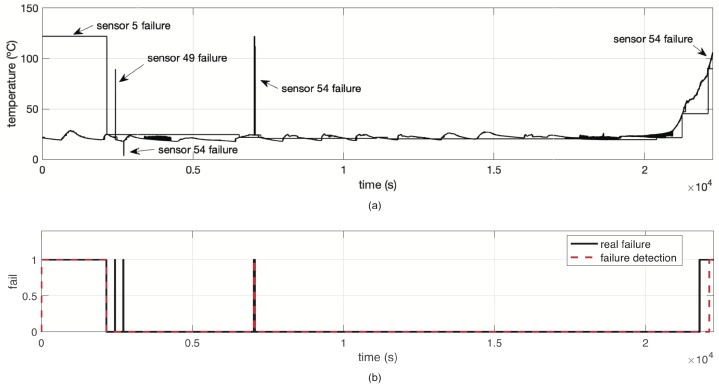
(**a**) Sensor failures in testing data and (**b**) failure detection response in the Intel Lab WSN with N=54 sensor nodes.

We applied the proposed SFDIA with AHN of Algorithm 2. After completion of the SFDIA strategy, this experiment reported an accuracy of 98.30% in failure detection phase. Figure 12 shows the testing data set with some failures, and the comparison between the estimated failure detection and the real failure. It can be observed that some regions of false-negative values are estimated. Unfortunately, the proposed SFDIA strategy cannot correct sensor failure detection in the latter regions. Nevertheless, this experiment validates the feasibility to detect sensor failures in an accurate way over real data.

## 5. Experimental Results and Discussion

We conducted a series of experiments over the WSN, described in Section 2, to evaluate the performance of our proposed SFDIA strategy based on AHN. Three enclosed temperature sensors ($s_{1},s_{2},s_{3}$) and a temperature remote web service ($s_{4}$) were considered as sensor nodes (N=4). We collected 2409 temperature samples from all the sensor nodes during ten days in a non-uniform way (no sample rate defined).

### 5.1. Training Phase

We considered the first seven days of data (1687 samples) as healthy information retrieved from the sensor nodes. So, these data were employed for training all the sensor and dynamic function AHN-models.

For the temperature sensor estimators, we configured the AHN models with the following parameters: molecules m=5, learning rate η=0.1 and a maximum of 1000 iterations as stop criterion. For the dynamic function estimators, the AHN models were configured with the parameters: molecules m=5, learning rate η=0.1 and a maximum of 1000 iterations as stop criterion. Notice that we only trained models related to the physical sensors in our WSN: $s_{1},s_{2}$ and $s_{3}$. For suitability, data were normalized uniformly before using for training the AHN-models.

**Figure 13:**
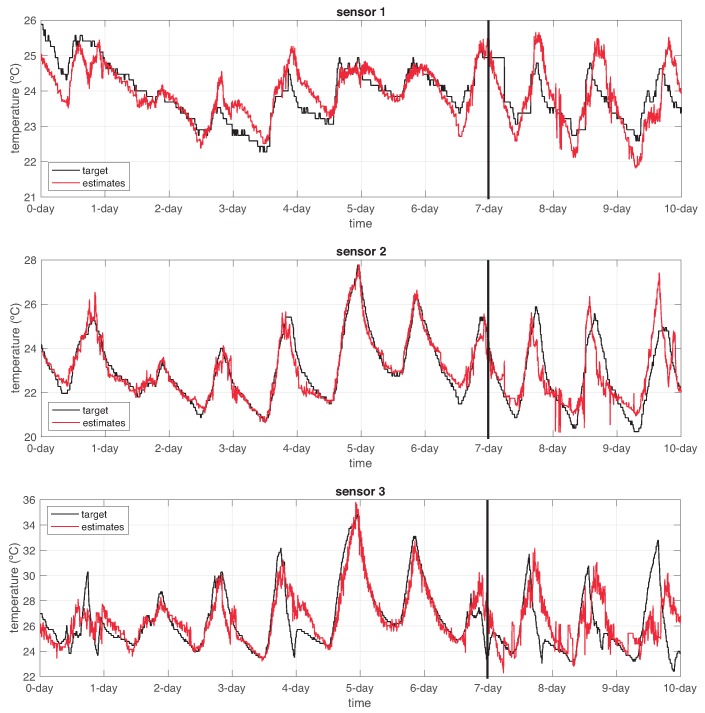
Temperature estimation associated to each physical sensor node $s_{1},s_{2}$ and $s_{3}$ in comparison with the actual values. Left side of the vertical line refers to training data and right side refers to the testing data.

Figure 13 shows the output response of the temperature sensor AHN-models. As observed, the left part depicts estimation over the training data and the right part depicts estimation over the testing data (722 samples representing three days). Table 2 summarizes the root-mean-squared error (RMSE) in the estimation for both training and testing in all the physical sensor nodes.

For the dynamic function estimators, we configured the AHN models with the following parameters: molecules m=5, learning rate η=0.1 and a maximum of 1000 iterations as stop criterion. For the dynamic function estimators, the AHN models were configured with the parameters: molecules m=5, learning rate η=0.1 and a maximum of 1000 iterations as stop criterion. In this case, we only trained models related to the sensor nodes: $s_{1},s_{2}$ and $s_{3}$. Notice that these models shape the dynamics of other sensor nodes, except for $s_{i}$. We normalized data uniformly before using for training the AHN-models.

**Figure 14:**
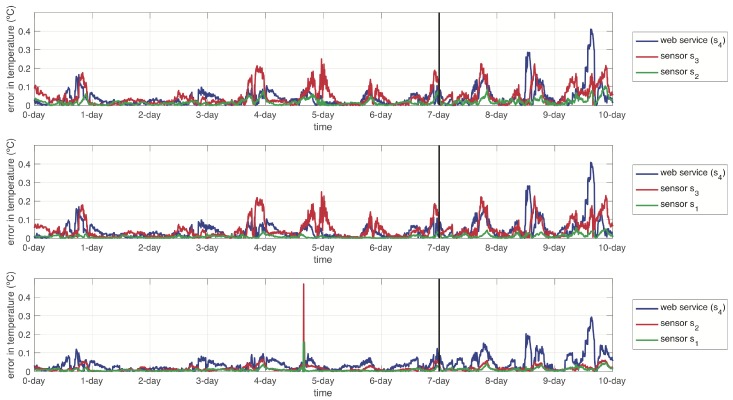
Absolute error between the dynamic function estimations associated to each sensor node $s_{1},s_{2}$ and $s_{3}$ in comparison with the actual values. Left side of the vertical line refers to training data and right side refers to the testing data.

Figure 14 shows the absolute error between the output response of the dynamic function AHN-models and the actual dynamics system. Again, the left part depicts estimation over the training data and the right part depicts estimation over the testing data. Table 3 summarizes the RMSE in the estimation for both training and testing in all the dynamic functions. Notice that these RMSE results are for each of the sensor node signals related in the dynamic function.

### 5.2. Failure Detection Phase

Using the above AHN-models, we conducted an experiment on sensor failure, so we can test and evaluate the performance of our SFDIA proposal. To do so, we collected data from the WSN in the three-day period (i.e., the testing set), and then we changed some sensor values to artificially produce sensor failures. The sensor failures where done by changing the real value with a random constant value below the mean values of sensors (measured in the training data). Then, we simulate an online failure detection.

**Figure 15:**
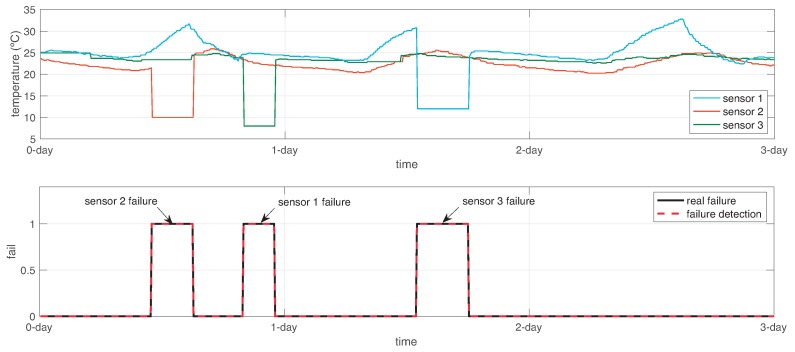
Failure detection response in the system.

At each simulation step, we computed the error between the dynamic function estimation ${\hat{f}}_{\omega }^{\left(i\right)}$ and the actual values of the sensors in WSN, except the sensor *i*, such that (10) is calculated. After that, we obtained the overall error by adding up the error in the dynamic function estimations, as expressed in (11). Lastly, we computed the failure function fail using (12) with a confidence value α=1.65 representing 95.05% of confidence in failure detection. Figure 15 shows the output response of this sensor failure detection. As observed, the failure detection is done in 100%, but until now, the sensor failed is not detected.

### 5.3. Sensor Identification and Accommodation Phases

**Figure 16:**
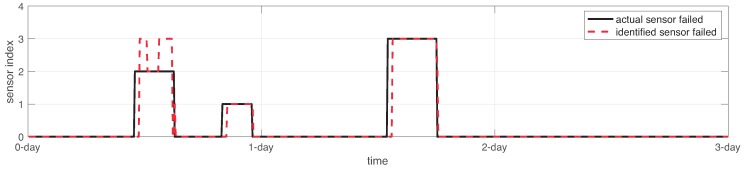
Sensor failure identification response in the system.

In order to identify the sensor who failed, the proposed SFDIA computes a comparison between the actual value of one sensor $s_{k}$ and the estimation provided by the sensor AHN-model ${\hat{s}}_{k}={\hat{fs}}_{\omega }^{\left(k\right)}$, as expressed in (13). Figure 16 shows the comparison between the actual and the estimated sensor failed. For this experiment, the accuracy of the sensor identification over the testing set was 94.18%.

**Figure 17:**
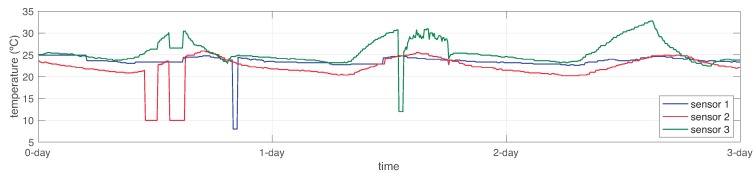
Sensor accommodation and final results over the WSN using the proposed SFDIA approach.

To this end, the sensor identification served for sensor accommodation. In this regard, failed values from sensors $s_{k}$ were changed by estimations from sensors ${\hat{s}}_{k}$, as shown in Figure 17. Notice that only 5.82% of testing data failed to be recovered.

### 5.4. Discussion

From the previous results, it can be seen that AHN-models can detect failures, identify and accommodate sensor nodes with accuracy over the testing set of 94.18%. In this context, the proposed method of intelligent failure detection to discover malfunctions has the following advantages: the response of the dynamic functions estimations in training and testing, using AHN-models proved high accuracy data; the system had the capability to detect the failure of sensor with a confidence of 95.05% in failure detection; in order to identify the sensor that fails, the proposed SFDIA provided a good accuracy in the detection response with 100% correct appreciation rate; furthermore, the sensor accommodation was obtained from this identification where only 5.82% of testing data failed to be recuperated.

Nonetheless, a number of limitations have been identified in the failure detection method that will need to be addressed in order to improve the efficiency in the system. It requires more data acquisition from sensor nodes in order to perform better accuracy to train the AHN-models, it needs more sensor nodes for failure detection in each one of them, and it also needs retraining to minimize the error in time and to collect healthy data. Also, it is important to highlight that the threshold parameter α was set experimentally. Thus, a further study on how to obtain this parameter is required. One possibility refers to compute α by simulating fake sensor failures during the training phase.

## 6. Conclusions

In this work, we proposed a sensor failure detection, identification and accommodation approach based on artificial hydrocarbon networks over a wireless sensor network. This approach consisted in two modules: the temperature estimation and the dynamic function models. In addition, an algorithm (c.f. Algorithm 2) was introduced as the implementation of this method.

After experimentation over large WSN with simulated and real data sets and a real small WSN, we observed that our proposal can detect sensor failures in a high accurate way. Moreover, sensor failure identification and accommodation could be done in 94.18% of accuracy over the testing period. This validates that our proposal using AHN can be used as an SFDIA over WSN.

For future work, we are considering extending our proposal for more sensors in the network as well as to provide real-time execution of the SFDIA based on AHN. We are also implementing this system in a real-world application for better analysis of our proposed SFDIA system. To this end, a study on obtaining the threshold parameter α is also required.

## Figures and Tables

**Table 1:** Accuracy in failure detection in different simulated WSN.

*N*	Accuracy (%)
20	92.13
50	88.09
100	88.03

**Table 2:** Output response of the temperature estimations in training and testing, using AHN-models.

Temperature Sensor Model	RMSE (${}^{\circ }$C) in Training	RMSE (${}^{\circ }$C) in Testing
${\hat{s}}_{1}$	0.4482	0.6986
${\hat{s}}_{2}$	0.4042	0.9739
${\hat{s}}_{3}$	1.1986	2.7036

**Table 3:** Output response of the dynamic functions estimations in training and testing, using AHN-models.

Dynamic Function Model	RMSE (${}^{\circ }$C) in Training	RMSE (${}^{\circ }$C) in Testing
${\hat{f}}_{\omega }^{\left(1\right)}$	(0.0196,0.0579,0.0403)	(0.0307,0.0838,0.1042)
${\hat{f}}_{\omega }^{\left(2\right)}$	(0.0087,0.0583,0.0404)	(0.0187,0.0824,0.1045)
${\hat{f}}_{\omega }^{\left(3\right)}$	(0.0097,0.0213,0.0352)	(0.0161,0.0225,0.0846)
